# Optimizing Multispecialty Preventive Care: Referral Patterns, Adherence, and Remission in Inflammatory Bowel Disease

**DOI:** 10.7759/cureus.102305

**Published:** 2026-01-26

**Authors:** Sarah A Bokaee, Anushka Deogaonkar, Maxwell S Madani, Marie L Borum

**Affiliations:** 1 Division of Gastroenterology and Liver Disease, Department of Medicine, George Washington University School of Medicine and Health Sciences, Washington, D.C., USA

**Keywords:** adherence to preventive measures, high-risk guidelines, multispecialty, preventive health care, referral patterns

## Abstract

Background

Extraintestinal manifestations of inflammatory bowel disease (IBD) frequently involve the skin and eyes. Although current management guidelines emphasize multidisciplinary surveillance, referral practices to dermatology and ophthalmology remain inconsistent across specialties, potentially affecting disease control and patient outcomes.

Methods

A retrospective chart review was conducted at the George Washington University Hospital, an urban tertiary academic medical center in Washington, D.C., USA, between August 2023 and August 2025. Adult patients (≥18 years) with Crohn’s disease, ulcerative colitis, or indeterminate colitis seen in the gastroenterology clinic were identified. Patients with active referrals to dermatology or ophthalmology were included, while those without documented referrals or with incomplete medical records were excluded. Referral patterns, adherence to specialty appointments, and remission status were analyzed. A total of 102 dermatology and ophthalmology referrals among 586 patients with IBD were evaluated.

Results

Adherence to both dermatology and ophthalmology referrals was significantly associated with disease remission (p < 0.05). Dermatology referrals demonstrated higher adherence rates and were more frequently preventive in nature, whereas ophthalmology referrals were more commonly initiated in response to active symptoms and disease complications.

Conclusions

Preventive dermatologic surveillance in patients with IBD is associated with improved adherence and favorable disease outcomes. This proactive model may inform the development of standardized referral frameworks across IBD-related specialties, particularly ophthalmology, to strengthen multidisciplinary care and optimize long-term patient outcomes.

## Introduction

Inflammatory bowel disease (IBD) is a chronic, systemic inflammatory disorder with manifestations beyond the gastrointestinal tract, affecting organs such as the skin and eyes. Extraintestinal manifestations are common and frequently involve the dermatologic and ophthalmologic systems. Ophthalmologic complications affect approximately 10%-43% of patients with IBD and range from episcleritis and uveitis to therapy-related cataracts and glaucoma [1-3]. Cutaneous involvement is similarly prevalent, with nearly one-third of patients with IBD developing skin manifestations, including inflammatory lesions and malignancies [4-6].

Because IBD commonly presents in early adulthood and requires lifelong management, preventive care and multidisciplinary surveillance are essential to reducing long-term morbidity and disease-related complications [7]. Patients with IBD often receive the majority of their medical care from gastroenterologists, with infrequent engagement with primary care providers, placing greater responsibility on subspecialty care coordination for health maintenance needs [7]. IBD is among the most prevalent chronic gastrointestinal conditions in the United States and is associated with substantial healthcare utilization and cost, underscoring the importance of effective preventive strategies to improve quality of life and disease-free remission [8].

Current IBD management guidelines emphasize multidisciplinary surveillance and preventive care; however, delivery of specialty-based preventive services remains inconsistent [7-9]. Prior studies have demonstrated that patients with IBD receive preventive services less consistently than the general population, often due to a focus on symptom control rather than surveillance, unclear delineation of provider responsibility, and logistical barriers to specialty care [7,8]. While existing literature outlines management of active dermatologic and ophthalmologic disease in IBD, limited data characterize referral patterns, adherence to specialty consultations, or how engagement with these services relates to disease outcomes, representing an important gap in multidisciplinary IBD care.

Dermatology and ophthalmology represent contrasting models of subspecialty engagement in IBD management. Preventive dermatologic surveillance has been incorporated into routine care for patients with chronic inflammatory and immunosuppressed conditions, particularly given the increased risk of melanoma and non-melanoma skin cancers in this population [6-9]. In contrast, ophthalmologic referrals in IBD are more commonly initiated in response to active ocular symptoms rather than as part of routine preventive screening [1-3]. Whether these differing referral paradigms translate into differences in referral adherence or clinical outcomes among patients with IBD has not been well characterized.

This study aimed to characterize dermatology and ophthalmology referral patterns and adherence among patients with IBD who received specialty referrals at a tertiary academic medical center and to examine associations between referral adherence and clinical remission. We hypothesized that dermatology referrals would more frequently be preventive in nature, whereas ophthalmology referrals would be reactive, and that adherence to either specialty referral would be associated with disease remission.

## Materials and methods

Study design and population

A retrospective chart review was conducted at the George Washington University Hospital, an urban tertiary-care academic medical center in Washington, D.C., USA. Adult patients (≥18 years) with a confirmed diagnosis of Crohn’s disease, ulcerative colitis, or indeterminate colitis who were evaluated in the gastroenterology clinic between August 2023 and August 2025 were identified. This study period was selected based on the availability and completeness of electronic health record (EHR) data following institutional implementation of a unified clinical documentation system.

Patients were included if they had at least one documented referral to dermatology or ophthalmology during the study period. Exclusion criteria included age <18 years, absence of a confirmed IBD diagnosis, lack of a documented specialty referral, or incomplete medical records precluding assessment of referral adherence or remission status. From a total cohort of 586 patients with IBD, 102 patients who received a dermatology and/or ophthalmology referral met the inclusion criteria and were analyzed. Each patient was treated as a single unit of analysis. When patients had more than one referral, the first qualifying referral during the study period was used for analysis.

Data collection and definitions

Demographic variables extracted from the EHR included age, age at IBD onset, sex, race, ethnicity, and number of gastroenterology clinic visits during the study period. Clinical variables included IBD subtype, disease duration, remission status at the time of referral, presence of extraintestinal manifestations, insurance type, and adherence to surveillance colonoscopy.

Referral adherence was defined as completion of the scheduled dermatology or ophthalmology appointment documented in the EHR. Remission status was determined by treating gastroenterologists based on clinical documentation and available endoscopic evidence. Standardized disease activity indices were not uniformly available and, therefore, were not used.

Referrals were classified as preventive or reactive through a structured chart review. Preventive referrals were defined as those placed for routine screening or surveillance (annual skin examinations or ophthalmologic screening), whereas reactive referrals were initiated in response to active symptoms, suspected pathology, or documented disease complications. Chart reviews were performed by trained reviewers under faculty supervision using a standardized abstraction approach. Reviewers received instruction on referral classification and data extraction from the EHR prior to review. Ambiguous cases were discussed with supervising investigators and resolved by consensus to ensure consistency in classification.

Statistical analysis

The primary unit of analysis was the patient. Continuous variables were compared using two-sample t tests or Mann-Whitney U tests, as appropriate based on data distribution. Categorical variables were analyzed using chi-square (χ²) tests or Fisher’s exact tests. Missing data were infrequent and handled using complete-case analysis. Statistical significance was defined as a two-sided p-value <0.05. All analyses were performed using R statistical software (R Foundation for Statistical Computing, Vienna, Austria).

## Results

Cohort overview

Among 586 patients with IBD, 61 (10.4%) were referred to dermatology and 41 (7.0%) to ophthalmology. No patients in the study received referrals to both dermatology and ophthalmology. To preserve a consistent unit of analysis, if any patients received multiple referrals, the first eligible referral during the study period would have been selected, and all analyses were performed at the patient level.

Dermatology referrals

Of the 61 dermatology referrals, 34 patients (55.7%) attended their consultation. The dermatology-referred cohort included 22 males and 39 females; 28 White, 29 Black, and four Asian patients. Diagnoses were ulcerative colitis (n=27), Crohn’s disease (n=33), and indeterminate colitis (n=1). Among the 34 patients who attended their appointments, 18 (52.9%) consultations were classified as preventive, primarily for routine annual skin examinations, while 16 (47.1%) were reactive, addressing specific cutaneous concerns such as precancerous lesions or dermatitis (Table 1). Preventive versus reactive classification was determined from dermatology consultation notes and could only be assigned for patients who attended their appointments.

**Table 1:** Reason for referral to dermatology due to suspected dermatoses related to inflammatory bowel disease

Reason for Dermatologic Referral	n
Precancerous lesions	6
Dermatitis	3
Prurigo nodularis	2
Psoriasis	1
Nevus biopsy	1
Acne	1
Perianal lesion	1
Dermatofibrosarcoma	1

Patients who attended dermatology visits were more frequently in clinical remission at the time of referral (97.0% vs 70.0%; Fisher’s exact p=0.005) and had a higher median number of gastroenterology clinic visits in the preceding two years (3.0 (IQR 1.1-4.9) vs 2.0 (IQR 1.0-3.0); Mann-Whitney p=0.017). No statistically significant differences were observed by age, sex, race, insurance type, IBD subtype, or colonoscopy adherence (all p≥0.10). These findings are descriptive, and while adherence was associated with remission, causal relationships cannot be inferred; it is plausible that patients in remission or more engaged in care were more likely to attend specialty visits.

Ophthalmology referrals

Among the 41 ophthalmology referrals, 17 patients (41.5%) attended their consultation. Adherent patients were older than non-adherent patients (mean 54.9 ± 13.6 vs 47.0 ± 13.5 years; t-test p=0.005). Clinical remission at the time of referral was more frequent among patients who attended their ophthalmology visit (100% vs 57.1%; Fisher’s exact p=0.002). No significant differences were observed by sex, race, insurance, colonoscopy adherence, age of IBD onset, or presence of extraintestinal manifestations.

Among the 17 adherent ophthalmology patients, three (17.6%) consultations were preventive, primarily for routine annual eye examinations, while the remaining 14 were reactive, addressing active ocular pathology, including glaucoma, uveitis, episcleritis, and conjunctivitis (Table 2). Preventive versus reactive classification could only be determined for patients who completed their appointments.

**Table 2:** Reason for referral to ophthalmology due to suspected ocular manifestations of inflammatory bowel disease

Reason for Ophthalmologic Referral	n
Glaucoma	3
Iritis/Uveitis	2
Episcleritis	2
Conjunctivitis	2
Cataracts	2
Dry eyes	1
Blepharitis	1
Nonspecific visual symptoms	1

Overall, these results demonstrate descriptive differences in adherence and referral patterns between dermatology and ophthalmology among patients who received referrals. Dermatology consultations were more frequently preventive among adherent patients, whereas ophthalmology consultations were largely reactive. While adherence was associated with higher rates of clinical remission, these associations are correlational, and unmeasured factors such as baseline health status and engagement in care may contribute. The study is limited by its retrospective, single-center design, small sample sizes, and reliance on clinical documentation for remission status, and findings cannot be generalized to the broader IBD population.

## Discussion

This study demonstrates distinct patterns of multidisciplinary specialty engagement among patients with IBD, with dermatology referrals more frequently reflecting a preventive care model and ophthalmology referrals remaining largely symptom-driven. While referral rates to both specialties were comparable, dermatology consultations were more commonly initiated for proactive surveillance, whereas ophthalmology visits were typically prompted by active ocular complaints. These findings highlight differences in specialty-specific care paradigms that may influence both referral intent and patient adherence.

Adherence to specialty referrals was associated with higher rates of clinical remission in both dermatology and ophthalmology, though these relationships are correlational and do not establish causality. It is plausible that patients in remission or with greater engagement in care are more likely to attend specialty appointments. These findings may reflect broader patient activation and adherence to recommended disease management strategies rather than a direct effect of referral attendance on disease control. Notably, adherence to surveillance colonoscopy did not differ between groups, suggesting that the observed associations were not solely attributable to general compliance with all aspects of IBD care. While few prior studies have examined adherence to non-gastroenterologic specialty referrals in IBD, our results are consistent with the literature demonstrating improved outcomes among patients who comply with preventive care measures [8].

The higher proportion of preventive dermatology referrals and the greater adherence observed are consistent with current guideline recommendations emphasizing routine skin cancer surveillance in patients with IBD, particularly those receiving immunomodulators or biologic therapies [7-9]. Preventive dermatologic care is well-defined and widely endorsed in contemporary IBD management frameworks, including the most recent American College of Gastroenterology clinical guideline update [9]. In contrast, ophthalmologic referral guidelines are less clearly defined, with most literature focused on managing active ocular manifestations rather than routine surveillance. This may contribute to the predominantly reactive nature of ophthalmology referrals observed in our cohort.

The reactive referral pattern in ophthalmology is notable given that ocular manifestations of IBD can parallel intestinal disease activity and, if untreated, may result in irreversible visual impairment. While our data do not allow causal inference, earlier identification of ocular complications through preventive or risk-stratified ophthalmologic evaluation could be beneficial, particularly for patients with known extraintestinal manifestations or severe disease phenotypes. A preventive framework similar to dermatology may enhance multidisciplinary coordination and better align ophthalmologic care with contemporary IBD management principles, but this remains a hypothesis to be tested in future studies.

Several limitations warrant consideration. This was a single-center, retrospective study, which restricts generalizability and precludes causal interpretation. Sample sizes, particularly in subgroup analyses (only 17 adherent ophthalmology patients, only three preventive ophthalmology visits), were small, limiting the reliability of p-values and comparisons. Referral intent and classification as preventive versus reactive were determined by chart review and may be subject to misclassification bias. Clinical remission was based on documentation and available endoscopic data rather than standardized disease activity indices. Finally, the literature on adherence patterns to multidisciplinary referrals in IBD is limited, especially for ophthalmology, which constrains direct comparison with prior studies. Despite these limitations, our findings highlight potential gaps in multidisciplinary preventive care and provide a hypothesis-generating framework to guide future research on standardized referral pathways in IBD.

## Conclusions

In this single-center retrospective study, dermatology referrals among patients with IBD were more frequently preventive in nature and demonstrated higher adherence compared with ophthalmology referrals, which were largely symptom-driven. Adherence to both dermatology and ophthalmology referrals was associated with clinical remission. However, this relationship should be interpreted as associative rather than causal, given the observational study design. These findings highlight differences in specialty-specific referral paradigms and suggest that structured preventive models, such as those commonly employed in dermatology, may represent a potential area for future investigation in other IBD-related specialties. While limited by sample size, retrospective design, and inclusion of referred patients only, this study provides a data-driven, hypothesis-generating comparison of multidisciplinary referral patterns in IBD and underscores the need for prospective studies to better define the role of preventive specialty engagement in comprehensive IBD care.
